# Receipt of mRNA COVID-19 vaccines preconception and during pregnancy and risk of self-reported spontaneous abortions, CDC v-safe COVID-19 Vaccine Pregnancy Registry 2020–21

**DOI:** 10.21203/rs.3.rs-798175/v1

**Published:** 2021-08-09

**Authors:** Lauren Head Zauche, Bailey Wallace, Ashley N. Smoots, Christine K. Olson, Titilope Oduyebo, Shin Y. Kim, Emily E. Peterson, Jun Ju, Jennifer Beauregard, Allen J. Wilcox, Charles E. Rose, Dana Meaney-Delman, Sascha R. Ellington

**Affiliations:** Centers for Disease Control and Prevention; Centers for Disease Control and Prevention; Centers for Disease Control and Prevention; Centers for Disease Control and Prevention; Centers for Disease Control and Prevention; Centers for Disease Control and Prevention; Centers for Disease Control and Prevention; Centers for Disease Control and Prevention; Centers for Disease Control and Prevention; National Institute of Environmental Health Sciences; Centers for Disease Control and Prevention; Centers for Disease Control and Prevention; Centers for Disease Control and Prevention

**Keywords:** pregnancy, spontaneous abortion, COVID-19 vaccine, pregnancy loss, miscarriage, vaccine safety, mRNA vaccine

## Abstract

**Background:**

There is continuing public concern about the safety of COVID-19 vaccination during pregnancy. While there is no compelling biological reason to expect that mRNA COVID-19 vaccination (either preconception or during pregnancy) presents a risk to pregnancy, data are limited. It is, however, well documented that SARS-CoV-2 infection during pregnancy is associated with severe illness and increased risk of adverse pregnancy outcomes. Among recognized pregnancies in high-income countries, 11–16% end in spontaneous abortion (SAB).

**Methods:**

People enrolled in v-safe, a voluntary smartphone-based surveillance system, who received a COVID-19 vaccine preconception or during pregnancy were contacted by telephone to enroll in the v-safe pregnancy registry. V-safe pregnancy registry participants who received at least one dose of an mRNA COVID-19 vaccine preconception or prior to 20 weeks’ gestation and who did not report a pregnancy loss before 6 completed weeks’ gestation were included in this analysis to assess the cumulative risk of SAB using Life Table methods.

**Results:**

Among 2,456 pregnant persons who received an mRNA COVID-19 vaccine preconception or prior to 20 weeks’ gestation, the cumulative risk of SAB from 6–19 weeks’ gestation was 14.1% (95% CI: 12.1, 16.1%). Using direct age standardization to the selected reference population, the age-standardized cumulative risk of SAB was 12.8% (95% CI: 10.8–14.8%).

**Conclusions:**

When compared to the expected range of SABs in recognized pregnancies, these data suggest receipt of an mRNA COVID-19 vaccine preconception or during pregnancy is not associated with an increased risk of SAB. These findings add to accumulating evidence that mRNA COVID-19 vaccines during pregnancy are safe.

## Introduction

Pregnancy increases the risk for severe COVID-19 illness, and COVID-19 during pregnancy is associated with increased risk of preterm birth and may be associated with increased risk for other adverse maternal and neonatal outcomes.^1–3^ Spontaneous abortion (SAB, often defined as pregnancy loss occurring from 6 to < 20 weeks’ gestation) is a common pregnancy outcome. Risk of SAB is highest in the first 12 weeks, substantially decreasing after the first trimester, and increases significantly with maternal age and co-morbidities.^4–7^ Among recognized pregnancies in high income countries, 11–16% result in SAB. ^4–10^

Initial data published from the v-safe pregnancy registry included 104 reports of SAB as of March 30, 2021.^11^ This report provides additional follow-up data on surviving pregnancies and SABs, in order to provide an estimate of risk. We estimate the cumulative risk of an SAB after receipt of an mRNA COVID-19 vaccine preconception or during pregnancy prior to 20 weeks’ gestation to assess whether the risk is in the range of previously reported background rates of SAB.

## Methods

### Study design

V-safe and the v-safe pregnancy registry have been described previously.^11–13^ Briefly, v-safe is a smartphone-based tool available for all people who have received a COVID-19 vaccination in the United States. Enrollment is voluntary, and participants complete web surveys which include questions about pregnancy status at the time of vaccination and since vaccination.^12^ Persons who report that they were pregnant at the time of vaccination or since vaccination and are 18 years or older are contacted by telephone and invited to enroll in the v-safe pregnancy registry. Enrolled participants receive a telephone follow-up each trimester, during the postpartum period, and three months following live births.^13^ This activity was reviewed by the Centers for Disease Control and Prevention (CDC) and was conducted consistent with applicable federal law and CDC policy; the activity met requirements of public health surveillance as defined in 45 CFR 46.102.^14^

### Participants

Data were analyzed from participants with a singleton pregnancy who received at least one dose of an mRNA COVID-19 vaccine preconception (30 days before the first day of the last menstrual period through 14 days after) or during pregnancy prior to 20 weeks’ gestation, and who had not reported a pregnancy loss before 6 completed weeks’ gestation. The inclusion of participants pregnant at 6 completed weeks’ gestation reflects when pregnancies are generally recognized and is consistent with previous literature estimating SAB in the general population.^5, 8–10, 15^

Given that the Janssen adenoviral vector COVID-19 vaccine is a different type of vaccine than mRNA COVID-19 vaccines, participants enrolled in the v-safe pregnancy registry who had received the Janssen vaccine (n = 272) were not included in this analysis. At this time, there are limited data available on pregnancy outcomes in these participants because the Janssen vaccine was granted Emergency Use Authorization (EUA) several months after the mRNA COVID-19 vaccines.

### Statistical Analysis

Descriptive analyses were performed. Gestational age at vaccination was calculated using the self- reported date of vaccination, and either self-reported estimated due date or date of the first day of the last menstrual period. When participants reported an SAB, the date of SAB was recorded based on clinical diagnosis when provided by the participant.

Life table methods were used to calculate the cumulative risk of SAB by gestational week.^8, 16^ Participants who received an mRNA COVID-19 vaccine in the preconception period or before 6 weeks’ of pregnancy were entered into the analysis at 6 weeks’ gestation whereas participants who received their first eligible dose at or after 6 weeks’ gestation entered the analysis in the week they received their first eligible dose. Participants who did not have contact with the v-safe pregnancy registry at or after 20 weeks’ gestation were censored at the time of last contact, and participants who reported other pregnancy outcomes (i.e., ectopic and molar pregnancies, induced abortions) were censored at the date of the outcome. The cumulative risk of SAB for a given gestational week was calculated by taking the product of the week-specific SAB risk and the cumulative risk of SAB at the preceding week for each week up to the week of interest. Log normal confidence intervals were calculated for cumulative risk of SAB at each gestational week. The cumulative SAB risk was also age-standardized using risk of SAB by age group from Magnus et al., 2019.^5^ Standard errors of the age-adjusted cumulative risk of SAB were estimated by dividing the age-adjusted rate by the square root of the number of SABs, and 95% confidence intervals were calculated.^17^

We conducted a sensitivity analysis to estimate the maximum possible risk of SAB in our cohort by assuming all participants who we were not able to contact in the second trimester experienced an SAB immediately after last contact. Statistical analyses were conducted using SAS software, version 9.4.

## Results

**Figure 1:**
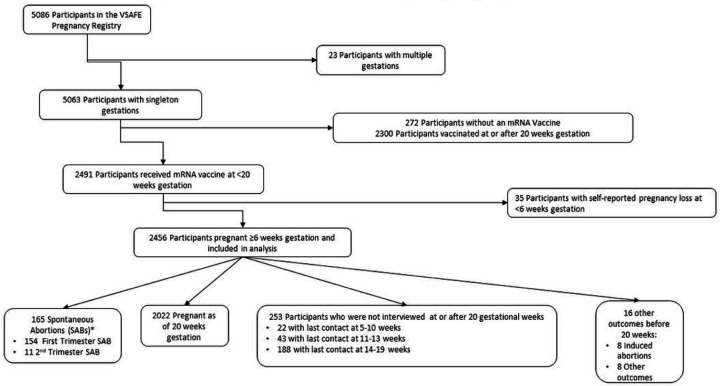
Inclusion Criteria: Receipt of mRNA COVID-19 vaccines preconception and during pregnancy and risk of self-reported spontaneous abortions, CDC v-safe COVID-19 Vaccine Pregnancy Registry 2020–21

As of July 19, 2021, 5,086 participants were enrolled in the v-safe pregnancy registry, of whom 5,063 reported a singleton pregnancy. Of these, 2,491 participants received at least one mRNA COVID-19 vaccine dose preconception or prior to 20 weeks’ gestation. A total of 35 participants self-reported a pregnancy loss (33 SAB and 2 ectopic pregnancies) at less than 6 weeks’ gestation and were excluded from the analysis. As a result, 2,456 people met criteria for inclusion as outlined in Figure 1.

The cohort consisted of mostly non-Hispanic white (78.3%) persons who identified as healthcare personnel (88.8%) with most participants aged 30–34 years (49.1%) or 35–39 years (28.2%) (Table 1). Among eligible participants, 27.5% had experienced at least one prior SAB and 9.1% had experienced at least two prior SABs. Over half of participants (52.7%) received the Pfizer-BioNTech vaccine and 47.3% received the Moderna vaccine. Date of first eligible vaccination ranged from December 14, 2020 through April 3, 2021, and 90% of participants received two doses of an mRNA COVID-19 vaccine.

Of the 2,456 participants in the cohort, 2,020 were known to be pregnant at 20 weeks’ gestation. The pregnancy status at 20 weeks’ gestation was unknown for 253 pregnant people (65 could not be contacted for second trimester follow-up and 188 completed second trimester follow-up before 20 weeks’ gestation). A total of 165 participants self-reported an SAB, of which 154 occurred prior to 14 weeks’ gestation.

The week-specific and cumulative risk of SAB from 6–19 weeks’ gestation along with their 95% confidence intervals are tabulated in Table 2. The cumulative risk of SAB from 6–19 weeks’ gestation was 14.1% (95% CI: 12.1–16.1%). When stratified by age, the cumulative risk of SAB from 6–19 weeks’ increased with increasing age: 9.8% (95% CI: 5.9–12.4%) among participants aged 20–29 years, 13.0% (95% CI: 10.1–15.8%) among participants aged 30–34 years, 16.7% (95% CI: 12.5–20.6%) among participants aged 35–39 years, and 28.8% (95% CI: 16.8–39.1%) among participants 40 years and older. Using direct age standardization to the selected reference population^2^, the age-standardized cumulative risk of SAB from 6–19 weeks’ gestation in the v-safe pregnancy registry was 12.8% (95% CI: 10.8–14.8%) (data not shown). In the sensitivity analysis, under the extreme assumption that all 65 participants with last contact in the first trimester experienced an SAB, the cumulative risk of SAB from 6–19 weeks’ gestation was 18.8% (95% CI: 16.6–20.9%) and was 18.5% (95% CI: 16.1–20.8%) after age standardization (data not shown). Age standardization had less of an effect on our sensitivity analysis than our primary analysis, because participants who were unable to be reached in the second trimester were younger than those with second trimester follow-up (data not shown).

## Discussion

While SARS-CoV-2 infection during pregnancy is associated with severe illness and adverse obstetrical outcomes^1–3^, COVID-19 vaccines were not expressly studied in pregnant people prior to availability in the United States under an EUA. While there is no compelling biological mechanism to expect that mRNA COVID-19 vaccines present a risk to pregnancy, data have been limited to guide pregnant people and healthcare professionals about vaccination in the preconception period and during pregnancy. In this report, we calculated the cumulative risk of SAB from 6–19 weeks’ gestation for people in the v-safe pregnancy registry who received an mRNA COVID-19 vaccine preconception or in pregnancy. These findings add to the accumulating evidence about the safety of COVID-19 vaccination in pregnancy.^11, 18^ Previous studies estimating cumulative risk of SAB in the general population have reported similar estimates as our study.^4–10^ However, the median age of our study population is higher than in the reference studies.^7–10^ Given that maternal age is a known risk factor SAB, this may have skewed our overall risk of SABs higher. When we age-standardized our estimate, the cumulative risk fell from 14.1% (95% CI: 12.1–16.1%) to 12.8% (95% CI: 10.8–14.8%). Consistent with previous studies that estimated week-specific SAB risk, our week-specific risk of SAB decreased after 12 weeks’ gestation.^8, 10^

Our sensitivity analysis resulted in an-age standardized risk of 18.5% (95% CI: 16.1–20.8% under the extreme assumption that all 65 pregnant people whom we were unable to contact during the second trimester experienced an SAB. It is possible that some of those not contacted in the second trimester did have an SAB, but it is highly unlikely that all did, especially given that these 65 participants were significantly younger than those with second trimester follow-up. Data in this report are preliminary, and active follow-up through the v-safe pregnancy registry is ongoing. Several limitations should be noted. First, the cohort does not include a comparison group of unvaccinated pregnant people. Additionally, the cohort is relatively homogenous with respect to racial and ethnic groups and occupation with 78% of participants non-Hispanic White and 89% healthcare personnel. Furthermore, data were collected both prospectively and retrospectively. Previous estimates of SAB proportions relied on prospective studies,^5,8–10^ in which pregnant people were enrolled before or at the start of pregnancy and followed over time to assess pregnancy outcomes, providing less biased estimates. Additionally, all data were self-reported, including vaccine administration dates, pregnancy status, estimated delivery date or gestational age, and pregnancy outcome. Given the voluntary nature of the registry, enrollment biases are likely. Pregnant people who experienced an SAB may have been more likely to enroll in the v-safe pregnancy registry, which would overestimate the risk. Inconsistencies in ability to obtain follow-up information may have also affected the incidence as some may not have been willing to complete their scheduled follow-up interviews if they experienced an SAB. Our sensitivity analysis accounted for that possibility.

Despite the limitations noted above and the inherent challenges of registry data without a comparison group, these data suggest that the cumulative risk of an SAB from 6–19 weeks’ gestation after receipt of an mRNA COVID-19 vaccine is within the expected range based on previous SAB studies. Confirmation of these results is needed from observational studies that include unvaccinated pregnant people. Our data as of July 19, 2021 are reassuring and do not suggest an increased risk of SAB following receipt of an mRNA COVID-19 vaccine in the preconception period or during pregnancy.

## Figures and Tables

*Percentage of SABs should not be calculated out of total participants included in the analysis and interpreted as a risk. See text for full analysis of SAB risk

**Table 1.** Pregnancy Status at 20 Weeks’ Gestation among Pregnant People Receiving mRNA COVID-19 Vaccines in the Preconception Period and through 20 Weeks’ Gestation, CDC v-safe COVID-19 Vaccine Pregnancy Registry: December 14, 2020—July 19, 2021

	All		Self-reported SAB* 6–19 weeks’gestation		Ongoing pregnancies^¶^ at 20 weeks’ gestation		Participants with ongoing pregnancy at contact prior to 20 weeks’ gestation		Other pregnancy loss 6–19 weeks gestation	
Characteristics	n	%	n	%^±^	N	%^±^	n	%^±^	n	%^±^
**Total**	**2456**		**165**		**2022**		**253**		**16**	
**Age at 1st eligible vaccine dose (years)**										
20–29	432	17.6	23	13.9	343	17.0	64	25.3	2	12.5
30–34	1205	49.1	71	43.0	1020	50.4	108	42.7	6	37.5
35–39	693	28.2	54	32.7	561	27.7	72	28.5	6	37.5
40+	126	5.1	17	10.3	98	4.8	9	3.6	2	12.5
**Race and Hispanic origin**										
Non-Hispanic Black	35	1.4	5	3.0	23	1.1	6	2.4	1	6.3
Non-Hispanic White	1923	78.3	113	68.5	1613	79.8	185	73.1	12	75.0
Hispanic	220	9.0	21	12.7	159	7.9	39	15.4	1	6.3
Non-Hispanic American Indian/Alaskan Native	7	0.3	0	0.0	6	0.3	1	0.4	0	0.0
Non-Hispanic Native Hawaiian/Pacific Islander	8	0.3	0	0.0	6	0.3	2	0.8	0	0.0
Non-Hispanic Asian	210	8.6	17	10.3	179	8.9	13	5.1	1	6.3
Non-Hispanic multiple races	48	2.0	7	4.2	34	1.7	6	2.4	1	6.3
Missing	5	0.2	2	1.2	2	0.1	1	0.4	0	0.0
**Self-reported vaccine priority group**										
Non-healthcare essential worker	148	6.0	6	3.6	110	5.4	30	11.9	2	12.5
Healthcare personnel^*†*^	2180	88.8	149	90.3	1822	90.1	196	77.5	13	81.3
**Vaccine type**										
Moderna	1162	47.3	85	51.5	944	46.7	126	49.8	7	43.8
Pfizer-BioNTech	1294	52.7	80	48.5	1078	53.3	127	50.2	9	56.3
**Number of vaccine doses reported**										
1	245	10.0	39	23.6	183	9.1	18	7.1	5	31.3
2	2211	90.0	126	76.4	1839	90.9	235	92.9	11	68.8
**Timing of Dose 1**										
Preconception**	380	15.5	56	33.9	212	10.5	106	41.9	6	37.5
First trimester (>=2 and <14 weeks)	1230	50.1	107	64.8	971	48.0	142	56.1	10	62.5
Second trimester (>=14 and <20 weeks^¶¶^)	846	34.4	2	1.2	839	41.5	5	2.0	0	0.0
**Timing of Dose 2 (n=2211)**										
Preconception**	188	8.5	19	11.5	116	5.7	50	19.8	3	18.8
First trimester (>=2 and <14 weeks)	885	40.0	91	55.2	642	31.8	145	57.3	7	43.8
Second trimester (>=14 and <28 weeks)	1125	50.9	4	2.4	1081	53.5	40	15.8	0	0.0
After pregnancy outcome	13	0.6	12	7.3	0	0.0	0	0.0	1	6.3
**Comorbidities and past medical history**										
Obesity^*±**±*^	432	17.6	34	20.6	340	16.8	57	22.5	1	6.3
Pre-existing diabetes	27	1.1	2	1.2	23	1.1	2	0.8	0	0.0
Prior SAB	675	27.5	59	35.8	549	27.2	65	25.7	2	12.5
*1 Prior SAB*	452	67.0	33	20.0	373	18.4	44	17.4	2	12.5
*2 or more Prior SABs*	223	33.0	26	15.8	176	8.7	21	8.3	0	0.0

* Spontaneous abortion

¶ Includes 19 live births, 5 stillbirths, 2 induced abortions after 20 gestational weeks, and 1996 pregnancies ongoing as of last interview

± Column percentages provide among each pregnancy outcome/status; these should not be interpreted as risk estimates

† Any person serving in a healthcare setting who has the potential for direct or indirect exposure to patients or infectious materials

** Preconception defined as 4 weeks before last menstrual period up to 2 weeks after

¶¶ Second trimester vaccination for dose 1 limited to receipt at less than 20 weeks’ gestation per study criteria

± 12 cases missing information to determine body mass index for obesity

**Table 2.** Risk of Spontaneous Abortion among v-safe Pregnancy Registry Participants, December 14, 2020—July 19, 2021

Gestational Age	Number at risk	Self-reported SAB*	Week-specific SAB* risk (%)	Cumulative SAB risk (%, 95% CI^¶^)
6.0	904	15	1.66	1.66 (0.83–2.48)
7.0	982	18	1.83	3.46 (2.30–4.61)
8.0	1032	37	3.59	6.92 (5.36–8.46)
9.0	1087	39	3.59	10.26 (8.44–12.04)
10.0	1118	19	1.70	11.79 (9.87–13.66)
11.0	1184	12	1.01	12.68 (10.72–14.60)
12.0	1274	9	0.71	13.30 (11.31–15.24)
13.0	1394	5	0.36	13.61 (11.61–15.57)
14.0	1534	0	---	---
15.0	1632	2	0.12	13.72 (11.71–15.68)
16.0	1742	2	0.11	13.81 (11.81–15.78)
17.0	1848	2	0.11	13.91 (11.90–15.87)
18.0	1941	3	0.15	14.04 (12.03–16.01)
19.0	2052	2	0.10	14.12 (12.11–16.09)

* Spontaneous abortion

¶ Confidence interval

## References

[1] Zambrano LD, Ellington SR, Strid P, Update: Characteristics of symptomatic women of reproductive age with laboratory-confirmed SARS-CoV-2 Infection by pregnancy status — United States, January 22–October 3, 2020. MMWR. 2020;69:1641–1647. 33151921 10.15585/mmwr.mm6944e3 PMC7643892

[2] Wei SQ, Bilodeau-Bertrand M, Liu S, Auger N. The impact of COVID-19 on pregnancy outcomes: a systematic review and meta-analysis. CMAJ, 2021; 193(16):E540–E548. 33741725 10.1503/cmaj.202604 PMC8084555

[3] Allotey J, Stallings E, Bonet M, Clinical manifestations, risk factors, and maternal and perinatal outcomes of coronavirus disease 2019 in pregnancy: living systematic review and meta-analysis. BMJ. 2020;370:m3320. 32873575 10.1136/bmj.m3320 PMC7459193

[4] Rossen LM, Ahrens KA, Branum AM. Trends in risk of pregnancy loss among US women, 1990–2011. Paediatr Perinat Epidemiol. 2018;32(1):19–29. 29053188 10.1111/ppe.12417 PMC5771868

[5] Magnus MC, Wilcox AJ, Morken NH, Weinberg CR, Haberg SE. Role of maternal age and pregnancy history in risk of miscarriage: prospective register based study. BMJ. 2019; 364. 10.1136/bmj.l869 PMC6425455 30894356

[6] Lang K, Neuvo-Chiquero A. Trends in self-reported spontaneous abortions: 1970–2000. Demography. 2012; 49(3): 989–1009. 22718315 10.1007/s13524-012-0113-0 PMC3787708

[7] Linnakaari R, Helle N, Mentula M, Trends in the incidence, rate, and treatment of miscarriage- nationwide registry-study in Findland, 1998–2016. Hum Reprod. 2019; 34 (11): 2120–2128. 31747000 10.1093/humrep/dez211

[8] Ammon Avalos L, Galindo C, Li DK. A systematic review to calculate background miscarriage rates using life table analysis. Birth Defects Res A Clin Mol Teratol. 2012;94(6):417–23. 22511535 10.1002/bdra.23014

[9] Wilcox AJ. Fertility and Pregnancy - An Epidemiologic Perspective, Oxford Univ Press, 2010. p 152

[10] Goldhaber MK, Fireman BH. The fetal life table revisited: Spontaneous abortion rates in three Kaiser Permenente cohorts. Epidemiology. 1991; 2(1): 33–39. 2021664

[11] Shimabukuro TT, Kim SY, Myers TR, Preliminary findings of mRNA Covid-19 vaccine safety in pregnant persons. N Engl J Med. 2021; 384: 2273–2282. 33882218 10.1056/NEJMoa2104983 PMC8117969

[12] Centers for Disease Control and Prevention. V-safe active surveillance for COVID-19 vaccine safety. January 2021. Accessed June 15, 2021. https:/www.cdc.gov/vaccinesafety/pdf/V-safe-protocol-508.pdf.

[13] Centers for Disease Control and Prevention. V-safe pregnancy surveillance (amendment). 2021. Accessed June 15, 2021. https:/www.cdc.gov/vaccinesafety/pdf/vsafe-pregnancy-surveillance-protocol-508.pdf.

[14] Department of Health and Human Services – 45 C.F.R. part 46, 21 C.F.R. part 56; 42 U.S.C. Sect. 241(d); 5 U.S.C. Sect. 552a; 44 U.S.C. Sect. 3501 et seq. Accessed June 17, 2021. https:/www.hhs.gov/ohrp/sites/default/files/ohrp/policy/ohrpregulations.pdf.

[15] Wang Conception, early pregnancy loss, and time to clinical pregnancy: a population-based prospective study. Fertility and Sterility. 2003; 79(3): 577–584. 12620443 10.1016/s0015-0282(02)04694-0

[16] Xu R, Chambers C. A sample size calculation for spontaneous abortion in observational studies. Reproductive Toxicology. 2011; 32: 490–493. 21907789 10.1016/j.reprotox.2011.08.009

[17] Keyfitz N. 3. Sampling variance of standardized mortality rates. Hum Biol. 1966;38(3):309–17. 5977534

[18] Goldshtein I, Nevo D, Steinberg DM, Association between BNT162b2 accination and incidence of SARS-CoV-2 infection in pregnant women. JAMA. Published online July 12, 2021. 10.1001/jama.2021.11035 PMC8276131 34251417

